# Recognizing Pinch Purpura as the First Manifestation of Light-chain Amyloidosis

**DOI:** 10.4274/tjh.2018.0016

**Published:** 2018-08-05

**Authors:** Erman Öztürk, Olga Meltem Akay, Burhan Ferhanoğlu

**Affiliations:** 1 Koç University Hospital, Clinic of Hematology, İstanbul, Turkey

**Keywords:** Amyloidosis, Purpura, Congo red

A 74-year-old female presented with a 6-month history of easy bruising as manifested by purpura after minor trauma to her face. Her physical examination was unremarkable except for the presence of pinch purpura scattered on her face (Figure 1). Laboratory tests showed leukocytes of 8100/µL, hemoglobin of 11.2 g/dL, platelets of 208,000/µL, prothrombin time of 11 s (normal range: 11.2-13.0 s), activated partial thromboplastin time of 25 s (normal range: 23.0-33.0 s), and erythrocyte sedimentation rate of 72 mm/h. Upon further workup, the presence of IgG lambda monoclonal gammopathy of the serum and lambda monoclonal light chain was found in urine immunofixation electrophoresis. Bone marrow biopsy revealed 7% lambda-restricted plasma cell infiltration, showing green birefringence with Congo red stain and vascular amyloid P deposition (Figure 2). There were no CRAB symptoms, organ dysfunction, or organomegaly. Echocardiography and pro-B-type natriuretic peptide results were normal. A diagnosis of amyloid light-chain (AL) amyloidosis initially presenting with purpura was made and a chemotherapy regimen of bortezomib and dexamethasone was started. Complete remission was achieved after six courses of chemotherapy and the purpuric lesions disappeared. 

Cutaneous manifestations are reported in 30%-40% of AL amyloidosis cases [1]. The lesions usually reflect capillary infiltration and fragility with petechiae and purpura, characteristically affecting the eyelids, beard area, and upper chest [2]. Purpura as the initial manifestation leading to the diagnosis of AL amyloidosis is relatively rare [3,4]. Therefore, cutaneous findings are valuable in making a diagnosis of this challenging disorder since early diagnosis before development of organ failure is essential for improving the prognosis of AL amyloidosis patients.

## Figures and Tables

**Figure 1:**
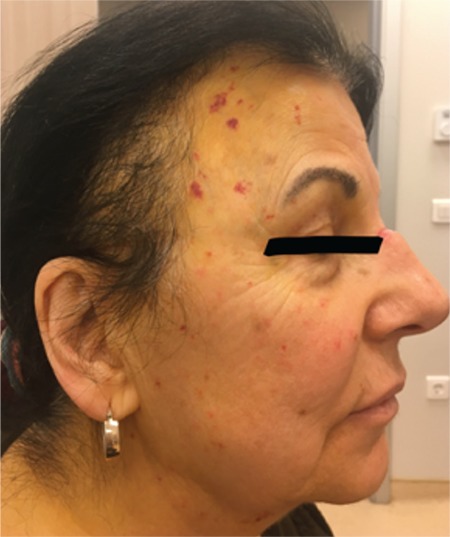
Purpura scattered on face (temporal region).

**Figure 2:**
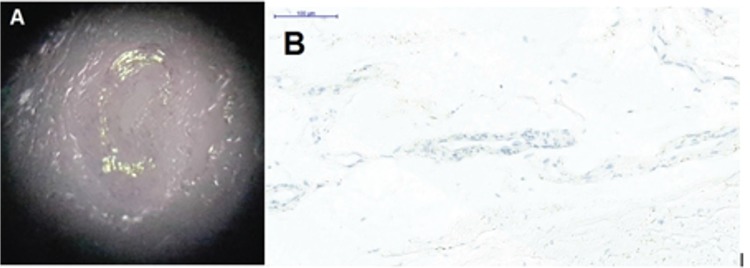
A) Microscopic section of the bone marrow stained with Congo red shows green birefringence under polarized light microscopy. B) Amyloid P with light microscopy.
